# Glans Resurfacing with Skin Graft for Penile Cancer: A Step-by-Step Video Presentation of the Technique and Review of the Literature

**DOI:** 10.1155/2019/5219048

**Published:** 2019-06-09

**Authors:** Athanasios Pappas, Ioannis Katafigiotis, Marjan Waterloos, Anne-Francoise Spinoit, Achilles Ploumidis

**Affiliations:** ^1^Department of Urology, Athens Medical Center, Athens, Greece; ^2^Urology Andrology Center of Piraeus, Greece; ^3^Department of Urology, Ghent University Hospital, Ghent, Belgium

## Abstract

**Introduction:**

Glans resurfacing has been suggested as a treatment option for the surgical management of superficial penile cancer (Tis, Ta, T1aG1, T1aG2). In this article we describe in detail the glans resurfacing technique with skin graft for penile cancer in a video presentation and we review the current knowledge of the literature.

**Material and Methods:**

The procedure is described in a stepwise fashion. Initially the patient is circumcised. The glans is marked in quadrants and completely stripped by dissecting and removing the epithelium and subepithelium layer of the glans. Deep spongiosal biopsies are taken to exclude invasion. Each quadrant is sent separately for biopsy. The surface of the graft size needed is estimated. A partial thickness skin graft is harvested from the thigh with a dermatome. The skin graft is then fenestrated. The graft is rolled over the glans and quilted with multiple sutures. A silicone 16F Foley catheter and a suprapubic catheter are placed. The penis is dressed with multiple gauzes and compressed with an elastic band.

**Results:**

The patient is discharged the next day. The dressing and Foley catheter are removed in 7 days. The patient continues to use the suprapubic catheter for 7 more days. The patient refrains from any sexual activity for 6 weeks and is closely followed.

**Conclusions:**

Glans resurfacing is an emerging new appealing surgical technique that is already a recommendation in the EAU guidelines for the treatment of premalignant and superficial penile lesions. The overall satisfaction rate and recovery of the sexual function are acceptable, and it can be considered an ideal procedure to treat superficial penile cancer.

## 1. Introduction

Penile cancer (PeCa), though uncommon, presents a geographical variation in incidence and a strong relation with the human papilloma virus (HPV) [1, 2]. One-third of the penile cancer cases are HPV-related, and even though the peak incidence is during the sixth decade of life, it can occur in younger patients [2]. Surgical management and the choice of infiltrative treatment depend on the stage and the invasiveness of the disease with penis sparing options reserved as a more overall suitable choice for the superficial disease up to T1a (TNM Classification) according to the European Urology Association (EAU) guidelines [3]. Radical surgical options, such as partial amputation and total penectomy, can impair sexual function or micturition, can affect genital sensibility, and can be detrimental for the psychology and the quality of life of the patient [3]. On the other hand, penile sparing surgery aims at complete excision of the primary tumor, with or without reconstruction, with the goal of preserving sexual function and improving cosmetic outcome [4]. Glans resurfacing is considered a new technique described since 2000 by Depasquale and was originally used for the treatment of extensive balanitis xerotica obliterans. Currently it has been suggested as a valid option for the surgical management of the superficial penile cancer (Tis, Ta, T1aG1, T1aG2) [4]. We describe in detail the glans resurfacing technique with skin graft for penile cancer in a video presentation (available here) and we review the current knowledge of the literature on the procedure.

## 2. Surgical Technique

### 2.1. Step 1: General Preparation

The patient is placed in a supine position, with the legs slightly abducted. The procedure is performed under general anesthesia. Broad-spectrum antibiotics are administered preoperatively. The external genital organs and the inner thigh are shaved in the operating room and surgical skin preparation is performed using antiseptic solution. The surgical field is draped leaving the genitals and the thigh exposed. Usually the right thigh is preferred for a right-handed surgeon standing on the right side of the patient.

### 2.2. Step 2: Circumcision and Tourniquet Appliance

**Figure 1:**
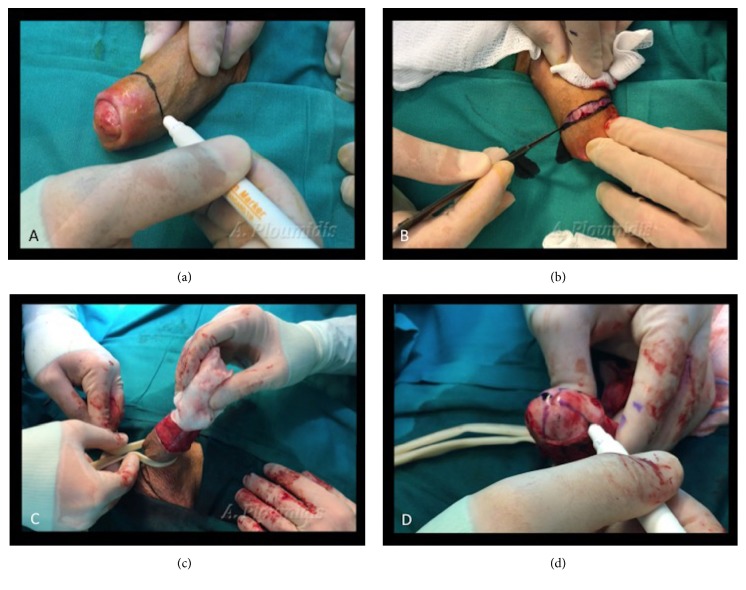
(a) Marking the circumcision line on the shaft of the penis. (b) Incising the marked skin, in order to perform the circumcision (sleeve technique). (c) Placing a tourniquet at the base of the penis. (d) The glans is marked in quadrants.

The prepuce skin is marked, just below the corona and on the shaft of the penis (Figure 1(a)). The excess foreskin is removed using the sleeve technique and the penis is circumcised (Figure 1(b)). Glans resurfacing begins by placing a tourniquet at the base of the penis (Figure 1(c)).

### 2.3. Step 3: Marking of the Glans in Quadrants and Dissecting of the Epithelium and Subepithelium Layer

**Figure 2:**
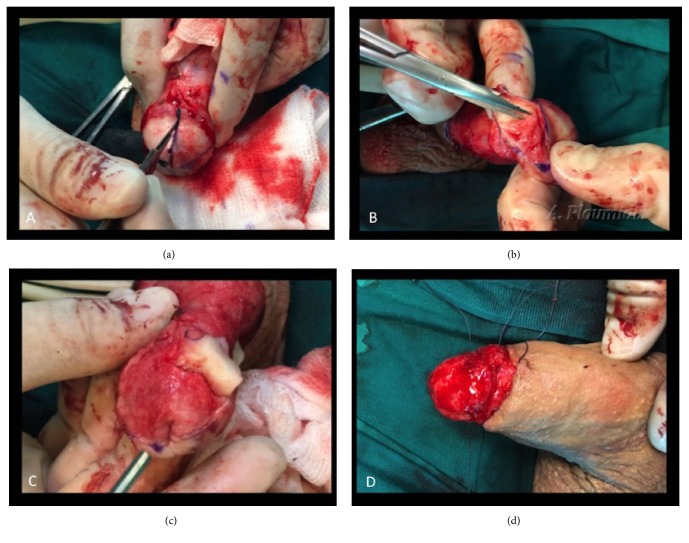
(a) Incising the epithelium and subepithelium of each quadrant of the glans. (b) Stripping the upper right quadrant of the glans, by removing the epithelium and subepithelium with tenotomy scissors (c) The upper right quadrant is completely peeled off. The upper left quadrant is semidetached and rolled backwards. The underlying spongiosum tissue is exposed. (d) The glans epithelium and subepithelium are completely removed.

The glans is marked in quadrants from the meatus to the corona (Figure 1(d)). The underlying spongiosum is exposed, by dissecting and removing the epithelium and subepithelium layer of the glans, from the meatus to the corona for each quadrant (Figure 2(a)). Dissection is undertaken with tenotomy scissors for better precision in dissection, or with a blade scalpel No 15 (Figure 2(b)). The plane of dissection lies between the thickened mucosa and the underlying spongy tissue. During the dissection the surgeon must take care that no affected mucosa is left on the glans especially near the meatus (Figure 2(c)). On the other hand, deep dissection down to the spongy tissue should be avoided not to cause unnecessary bleeding or to compromise the aesthetic outcome. The glans is completely stripped when all quadrants are removed and deep spongiosal biopsies can be taken to exclude invasion (Figure 2(d)). Each quadrant is sent separately for biopsy. After releasing the tourniquet compression, excessive bleeding is controlled with bipolar diathermy. Excessive cautery should be refrained in order to avoid necrotic tissue to the graft bed.

### 2.4. Step 4: Estimating Graft Size, Harvesting and Placement of the Skin Graft

**Figure 3:**
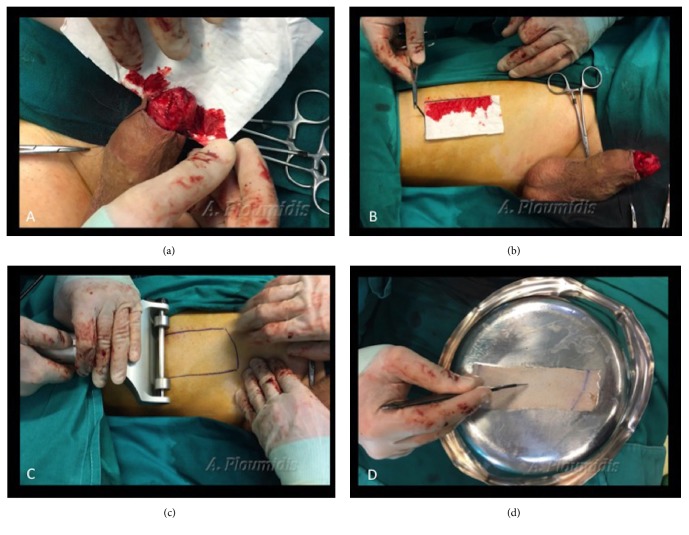
(a) Estimating the graft size needed by accurately placing a white paper over the glans circumference. Blood is absorbed from the paper defining the borders. (b) Marking the size of the skin graft over the harvesting site of the thigh taking into account graft contraction once removed. (c) A dermatome is used for the harvesting of the partial thickness skin graft. (d) Perforating the skin graft, in order to improve graft survival.

**Figure 4:**
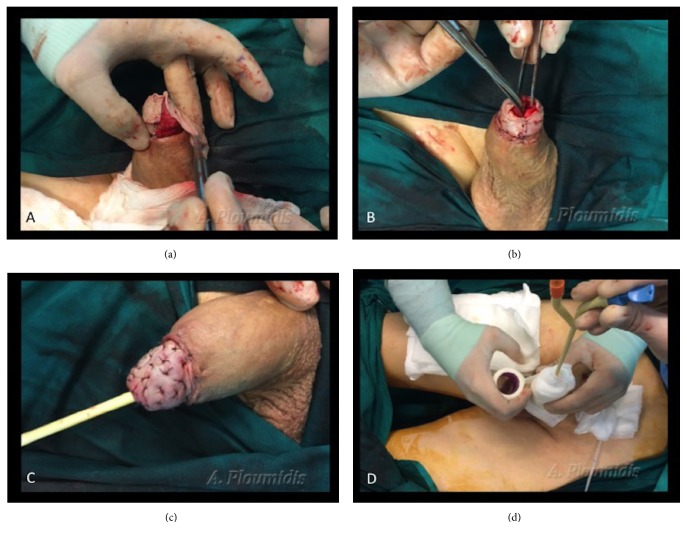
(a) The graft is rolled over glans starting from the ventral side. Quilting sutures are placed accordingly. (b) A meatotomy is performed to compensate for possible stricture at the level of the meatus due to sutures approximating the skin graft to the urethra. (c) The proximal end of the graft is sutured to the distal shaft skin by everting the edges in order to recreate the corona of a normal penis. Multiple quilting sutures secure the graft to its bed. (d) A suprapubic and a urethra catheter are placed. The penis is dressed with multiple gauzes and compressed with an elastic band for graft immobilization.

The surface of the graft size needed is estimated by accurately placing a white paper over the glans circumference. Blood is absorbed from the paper defining the size of the graft bed (Figure 3(a)). The paper is placed over the harvesting site, on the front or inner part of the thigh and the borders of the graft are marked taking into account graft contraction once removed (Figure 3(b)). A partial thickness skin graft is harvested from the thigh with a dermatome. The graft thickness ranges from 0,02 to 0.04 cm and is carefully trimmed (Figure 3(c)). The surgeon perforates the skin graft with a scalpel blade to allow blood and fluid accumulation to drain, thus improving graft survival (Figure 3(d)). The graft is rolled over glans starting from the ventral side (Figure 4(a)). Quilting sutures (5-0 absorbable polyglactin) are placed over the glans to secure the graft to its bed. In cases where a circumcision is performed due to the prepuce skin either being part of the disease or being strongly adhered to the glans, the proximal end of the graft is anastomosed with the distal shaft skin by everting the edges in order to recreate the corona sulcus (Figure 4(c)). A meatotomy is performed to compensate for possible stricture at the level of the meatus since the skin graft is inverted and approximated directly to the urethra (Figure 4(b)).

### 2.5. Step 5: Wound Dressing, Urinary Diversion, and Follow-Up

A silicone 16F Foley catheter and a suprapubic catheter are placed to prevent urine extravasation to the graft. The penis is dressed initially with a paraffin gauze and further covered with multiple gauzes and compressed with an elastic band (Figure 4(d)).

The patient is discharged the next day with instructions for bed rest for the following two days for better immobilization of the graft. The dressing and Foley catheter are removed in 7 days, and the graft is observed for inflammation or any necrotized tissue. The patient continues to use the suprapubic catheter for urine evacuation in order to keep the graft dry for 7 more days. The patient is advised to avoid any friction to the graft and refrain from sexual intercourse for 6 weeks. The patient is examined on a 3-monthly basis for 2 years, on a 6-monthly basis for a further two years, and then annually.

## 3. Discussion

PeCa diagnosis has a detrimental impact on the psychology of the patient, not only because a cancer has been diagnosed, but also due to the fact that the surgical management of the disease can affect adversely the cosmetic penile appearance, the sexual function, and the micturition of the patient [4]. The mainstay of surgical management is based on wide excision with partial or total penectomy involving the glans and the corpora cavernosa in cases of invasive PeCa [5, 6]. However, the difficulty patients have in accepting the amputative results of radical operations led to the emergence of new reconstructive surgical techniques with organ sparing orientation, not only for premalignant or superficial lesions but also for more advanced tumors. This approach aims to preserve the phallus and improve quality of life without compromising the oncological result [5, 6].

Glans resurfacing is considered a new technique in the field of reconstructive urology and it has been originally used for the treatment of severe lichen sclerosis of the glans [4]. Currently it is recommended from the EAU guidelines as a primary option for the management of the superficial noninvasive disease (PeIN) or as a secondary option after failure of topical chemotherapy or laser therapy. Furthermore, it is also recommended as a primary option for the management of superficial Ta, T1a (G1, G2) tumors [3].

The major studies referring to glans resurfacing technique with a median follow-up of at least 30 months reported no cancer-specific deaths, while the rates of the local recurrence fluctuated between 0 and 6% [3–7]. Glans resurfacing technique can be total (TGR) or partial (PGR) (excision of less than 50% of glans epithelium) [3, 4]. PGR is usually indicated in cases of localized CIS affecting less than 50% of the glans. During PGR only glans epithelium and subepithelium of the locally affected area are dissected with a macroscopic clear margin [3]. The TGR technique has been described in detail in previous studies referring to the procedure but has never been presented as a step-by-step technique in a video presentation [3, 6–8].

After performing the circumcision, the first important step is the exposure of the spongiosum by removing the epithelium and subepithelium tissue of the glans, from the meatus to the corona for each quadrant (Step 3) [3, 6, 7, 9]. The significance of positive surgical margins in organ sparing surgery for PeCa is of utmost importance; thus each removed quadrant is sent separately for frozen section. Furthermore, a crucial part of this stage of the procedure is deep spongiosal biopsies acquisition, in order to confirm the absence of any invasion [3, 7, 9]. In case of a positive surgical margin, adjuvant treatment or surgical excision can be performed, although some authors have proposed following-up the patient in case of PeIN [3, 7]. It is important to stress that for the TGR technique the positive surgical margins (PSM) have been reported to be up to 20%, while when taking into consideration both the PGR and the TGR, the PSMs can reach 45% [3, 4]. Even though the overall recurrence rate was only 4%, the rate of secondary operation after performing TGR was 10% [3, 10]. Glansectomy was the preferred technique as a second procedure in most of the cases [3, 8, 9].

The next important stage of the operation is the estimation of the graft size, the harvesting and the “transplantation” of the selected graft (Step 4). Although graft-rejection in reconstructive operations is always a possibility, in TGR graft failure is not considered common [3, 6, 7]. The reduced rate of graft failure and the need for regrafting can be attributed to the increased vascularity of the underlying corpus spongiosum [5, 7]. In one study of 17 patients, two of them showed partial graft loss and wound separation that were managed successfully with conservative treatment [6]. Only in one case in a series of 25 patients, the graft failed to heal completely and the patient was submitted to a secondary glansectomy. The histology in this particular patient was an invasive grade II pT1 SCC [3].

A few key steps during this part of the operation can increase the success rate of inosculation. When the tourniquet is released, small bleeding vessels of the spongiosum should be coagulated meticulously and with accuracy in order to avoid any underlying hematoma, but at the same time taking care of the normal vascularity of the graft bed. Moreover, it is important to harvest a partial thickness graft ranging from 0,02 to 0.04 cm, carefully trimmed to fit and quilted with interrupted sutures in order to promote graft survival. Some authors have proposed immobilization of the skin graft without quilting but instead covering the neoglans with proflavine soaked gauge dressing anchored with tie-over sutures (TODGA-technique) [10]. Furthermore some surgeons suggest performing multiple small incisions (fenestration) on the graft to allow mild exudate to drain in order to prevent any underlying hematoma or seroma formation (Step 4) [6].

The procedure is completed with the placement of a Foley catheter and a compressive dressing to the penis [1–7]. We also recommend a placement of a suprapubic catheter in order to divert the urine when the Foley catheter is removed. This is especially performed in cases where PeCa is over the meatus and the excision of the glans epithelium leaves the urethra exposed. In this case, the graft healing can be compromised due to the urinary stream on the urethra-graft anastomosis at the fossa navicularis.

Generally the procedure is associated with minimal risk of intraoperative and postoperative complications like bleeding, infection, and hematoma [4, 5, 7]. The recovery of the patient is rapid, cosmetic result is acceptable, the restoration of glans sensation is expected to be prompt, and penile function is preserved [4, 6, 7, 11–13]. In the main studies for the TGR all the patients that were preoperatively sexual active regained the same sexual activity within 3-5 months [6, 7]. However, it is vital that the patients are informed about the possibility of a positive surgical margin and the potential need for a secondary auxiliary procedure [5].

## 4. Conclusions

Glans resurfacing is an emerging new appealing surgical technique that is already a recommendation in the EAU guidelines for the treatment of premalignant and superficial penile lesions. It is important to carefully select, harvest, and suture the graft to the recipient bed of the glans. Negative surgical margins are of great importance for oncological reasons and to avoid possible auxiliary procedure. The overall satisfaction rate and recovery of the sexual function are acceptable, and it could be considered an ideal treatment for the superficial penile cancer.

## Supplementary Materials

Supplementary Materials Video presentation of a step-by-step surgical technique on glans resurfacing for penile cancer.
